# Supplementary material for: Feasibility Assessment of the Let’s Walk Programme (CAMINEM): Exercise Training and Health Promotion in Primary Health-Care Settings
Source: Int J Environ Res Public Health. 2021 Mar 19;18(6):3192. doi: 10.3390/ijerph18063192 (PMC8003347; doi:10.3390/ijerph18063192)
Supplement: Supplementary file 1 [file ijerph-18-03192-s001.zip › Figure S1_Referral procedure.docx]

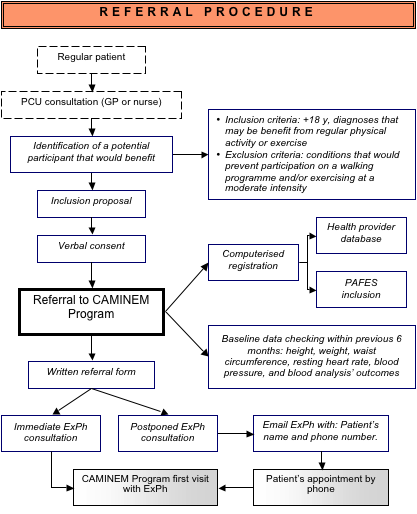


Figure S 1. CAMINEM Referral procedure.

*Note*. PCU = primary-care unit, GP = general practitioner, y = years, PAFES = Catalan plan for physical activity, sport and health, ExPh = Exercise Physiologist.

Broken-line box = actions done by the participant, italics box = actions done by the health practitioner, grey box = actions done by the exercise physiologist.
