# Supplementary material for: Feasibility Assessment of the Let’s Walk Programme (CAMINEM): Exercise Training and Health Promotion in Primary Health-Care Settings
Source: Int J Environ Res Public Health. 2021 Mar 19;18(6):3192. doi: 10.3390/ijerph18063192 (PMC8003347; doi:10.3390/ijerph18063192)
Supplement: Supplementary file 1 [file ijerph-18-03192-s001.zip › Figure S4_Discharge procedure.docx]

Figure S 4. CAMINEM Discharge procedure.

*Note.* min = minutes, wk = week, PCU = primary care unit.

Broken-line box = actions done by participants, grey box = actions done by the ExPh, green box = positive outcome, red box = negative outcome.
