# Supplementary material for: Feasibility Assessment of the Let’s Walk Programme (CAMINEM): Exercise Training and Health Promotion in Primary Health-Care Settings
Source: Int J Environ Res Public Health. 2021 Mar 19;18(6):3192. doi: 10.3390/ijerph18063192 (PMC8003347; doi:10.3390/ijerph18063192)
Supplement: Supplementary file 1 [file ijerph-18-03192-s001.zip › Figure S5_Progression criteria.docx]

| **TABLE S.5 Criteria to determine exercise prescription progression** | |
| --- | --- |
| **PREVIOUS EXERCISE PRESCRIPTION FULFILMENT** | **NEW EXERCISE PRESCRIPTION** |
| **TYPE – URBAN ROUTES** |  |
| Yes | Keep the route(s). |
| No | Discuss route changes, try other urban routes. |
| **VOLUME – FREQUENCY** |  |
| Yes – Equal or one day less | Keep frequency, discuss to increase one more day |
| No – Less than one day | Discuss route changes, reinforce and motivate. |
| **VOLUME – DURATION**  **INTENSITY – SELF-REPORTED** |  |
| Yes – Time previously set or ±10min   1. High (Vigorous) 2. Moderate 3. Low (Light) | 1. Increase duration. Encourage to focus on the talk test rather than time. 2. Keep duration, or discuss decreasing it (speed up). 3. Discuss decreasing duration (speed up). |
| No – Self-reporting >10min   1. High (Vigorous) 2. Moderate 3. Low (Light) | 1. Increase duration (slow down). Encourage to focus on the talk test rather than time. 2. Increase duration (slow down), as participant self-reports. 3. Keep duration, encourage speeding up. |
| No – Self-reporting <10min   1. High (Vigorous) 2. Moderate 3. Low (Light) | 1. Keep duration. Encourage to focus on the talk test rather than time. 2. Decrease duration (speed up), as participant self-reports. 3. Decrease duration (speed up) or double lap, adapting total duration. |
| **PROGRESSION AND ROUTINE** | **GOAL** |
| Adherence to previous prescriptions  Conditioning period (0 to 2 months)  Improvement period (2 to 6 months)    Maintenance period  Not adherence (empty logbooks)  Not retention (regular absences) | To achieve 150min·wk^-1^ of moderate-intensity aerobic exercise. Follow-up face-to-face meetings each three weeks.  To increase volume, first frequency then duration. Discuss more routes inclusion. Follow-up face-to-face meetings each eight weeks.  To maintain or increase volume. Discuss double laps and more routes inclusion. Follow-up face-to-face meetings each twelve weeks.  To increase adherence. Follow-up face-to-face meetings each three weeks.  To increase retention. Follow-up face-to-face meetings each three weeks. |
| *Note: min = minutes, wk = week* | |
